# The correlations between dynamic contrast enhanced magnetic resonance imaging and immunohistochemical data in head and neck squamous cell carcinomas

**DOI:** 10.55730/1300-0144.5543

**Published:** 2022-09-21

**Authors:** Nuri KARABAY, Hande Melike BÜLBÜL, Ersoy DOĞAN, Ahmet Ömer İKİZ, Göksenil BÜLBÜL, Sülen SARIOĞLU

**Affiliations:** 1 Department of Radiology, School of Medicine, Dokuz Eylül University, İzmir, Turkey; 2 Department of Otorhinolaryngology, School of Medicine, Dokuz Eylül University, İzmir, Turkey; 3 Department of Pathology, School of Medicine, Dokuz Eylül University, İzmir, Turkey

**Keywords:** Head and neck region, squamous cell carcinoma, magnetic resonance imaging, dynamic contrast enhancement, immunohistochemical data

## Abstract

**Background/aim:**

Dynamic contrast enhanced magnetic resonance imaging (DCE-MRI) can in vivo characterize tumor microvascular environment. The aim of the present study was to reveal the DCE-MRI findings and to determine the correlation between these findings and immunohistochemical data in head and neck squamous cell carcinoma (HNSCC).

**Materials and methods:**

Thirty-three patients diagnosed with primary HNSCC were evaluated retrospectively. DCE-MRI was conducted in all cases. CD34, CD105, and ki-67 expressions were analyzed with immunohistochemistry in tissue sections to determine micro-vessel density and proliferative activity.

**Results:**

The DCE-MRI is a successful technique in distinguishing tumor tissue from normal tissue. It was determined that Ve, Ktrans, and ki-67 values were significantly higher in high-stage tumors and there were positive correlations between the Ktrans value (by standard ROI) and CD34 MVDmax and CD34 MVDmean values. No statistically significant correlation was determined between other parameters in DCE-MRI and immunohistochemical data, and T stage.

**Conclusion:**

DCE-MRI could successfully differentiate tumor tissue in HNSCC. Furthermore, it was observed that DCE-MRI had the potential to reveal certain immunohistochemical information in vivo.

## 1. Introduction

In head and neck squamous cell carcinoma (HNSCC), the tumor stage is associated with nodal and distant metastases, local recurrence, and total survival [1–3]. However, since the biological behavior of HNSCC is variable, patients in the same tumor stage may have a different prognosis or may give dissimilar responses to the same treatment [2,3]. Thus, anticipating the prognosis and treatment response based on the tumor stage alone may not be sufficient. There is no biomarker in HNSCC with the sensitivity or specificity to predict histopathological information during the preoperative period. Angiogenesis is a significant factor in tumor growth, and it is vital to observe and measure growth quantitatively to determine the malignant behavior of the tumor [4]. The quantitative analysis of angiogenesis could be performed using immunohistochemical methods and antibodies specific to the antigens found in vascular endothelium, such as CD34 and/or CD105 [5,6]. Although there are certain limitations, positron emission tomography can provide practical information, while perfusion imaging can provide information on capillarity; however, both methods lack adequate histopathological details [1,7]. However, dynamic contrast enhanced magnetic resonance imaging (DCE-MRI) can in vivo characterize tumor microvascular environment [7,8]. It has been shown in the literature that these techniques provide data on benign-malignant tumor differentiation and tumor immunohistochemistry and predict tumor response to treatment in various malignancies such as the rectum, breast, prostate, and glial tumors [4,7–10]. Similar studies were conducted on HNSCC; however, the number of publications and reported information was insufficient [1,4,7,11].

The objective of the study was to calculate the correlation between the parameters of DCE-MRI and the immunohistochemical findings in HNSCC.

## 2. Materials and methods

### 2.1. Patients

Thirty-six patients diagnosed with primary HNSCC between December 2018 and June 2020 in our clinic and who underwent DCE-MRI were evaluated retrospectively. Three patients were excluded from the study due to inadequate MRI images. Thirty-three patients (6 women, 27 men; mean age 61.9, age range: 22–94) were included in the study. Fifteen patients had a biopsy and 18 had both a biopsy and an excision (underwent surgical resection). DCE-MRI was conducted before tissue samples were collected (average 19 days, range: 1–30 days). Exclusion criteria included 1) patients younger than 18 years, 2) inadequate DCE-MRI images due to artifacts, 3) tumor treatment prior to DCE-MRI, 4) lack of tissue samples for immunohistochemical evaluation, and 5) the presence of concomitant cancers in different anatomical regions.

Tumor localization in the oral cavity was observed in thirteen patients, in the larynx in eleven patients, and in the oropharynx in nine patients.

For statistical purposes, the cases were divided into two groups based on the TNM staging system. Patients in T1 and T2 stages were included in Group 1 (N = 15) and patients in T3 and T4 stages were included in Group 2 (N = 18).

The MR examination was conducted with a 1.5 T MR scanner (Achieva, Philips Medical Systems, Netherlands) with a head-neck coil.

### 2.2. DCE-MRI parameters

DCE imaging was performed using the T1W DCE sequences (T1W Single Shot Turbo Field Echo, matrix 256 × 256). Before the DCE-MRI sequence, T1 mapping was conducted with two flip angles of 5° and 15° (TR:10 ms and TE:2.4 ms, axial plane, section thickness: 3 mm). T1W DCE sequence that included 50 subsequent scans in 6 s was conducted before, during, and after administering an intravenous contrast agent with gadolinium. Gadoterat Meglumin (Dotarem, Guerbet Medical Supplies and Devices Pharmaceutical Industry and Trade Co.) was applied as a contrast agent at a dose of 0.2 mmol/kg and at the rate of 2 mL/s intravenously in the forearm with an automatic injector and then 20 mL saline was injected.

### 2.3. Analysis of the images

**Figure 1:**
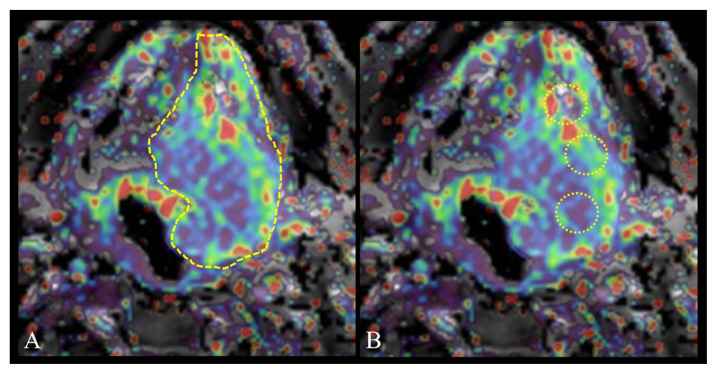
Schematic drawing of the widest ROI (A) and the standard ROI (B) in Ktrans map.

All images were transferred to a software module (IntelliSpace Portal-v8.2.20820, Philips Medical Systems, Netherlands) and all measurements were conducted using the workstation. The Ktrans, Kep, Ve, and iAUC [Ktrans = volume transfer constant (10^−3^/dk), Kep = rate constant reflects the backflow from the tumor to the vascular system, Ve = extravascular extracellular space volume ratio (10^−3^), iAUC = initial area under the curve] values were calculated with the Tofts Model, based on population-averaged AIF [12]. Two different 2D ROI (region of interest) measurements were conducted in quantitative analysis. 1) Widest section ROI: free-hand ROI drawn in the section where the tumor was the widest, avoiding necrosis areas using conventional images (Figure 1A). 2) Standard ROI: in lesions larger than 1 cm, three nonintersecting ROIs of 0.5 cm^2^ were drawn and the average of the 3 values was calculated, while in lesions smaller than 1 cm, a single 0.5 cm^2^ ROI was placed in the most solid area (Figure 1B). The normal tissue measurement was obtained with ROI placed in normal tissue symmetric to the lesion. Two radiologists (ten years and five years’ experience in head and neck oncology), blind to all clinical data, analyzed the images. The location of the ROI was determined by the joint decision of the two radiologists. Two measurements were made from the same location and the average of the measurements was used for statistical processes.

### 2.4. Immunohistochemical analysis

Immunohistochemical staining of the tissue sections in paraffin-embedded blocks was conducted, as described previously, with ki-67 (Roche Diagnostics., prediluted), CD34 (Roche Diagnostics., prediluted), and CD105 (Cornomics, 1/100 dilution) primary antibodies [6]. Digital images of the sections were evaluated on a computer via a camera attached to a light microscope. The number of micro-vessels with CD34 and CD105 antibodies was determined in 5 hotspots in the sections available at ×10 magnification on the computer screen individually. For the five regions, the value of the maximum count in one region and the mean of the counts in five regions were used in the statistical analysis. Ki-67 index was analyzed with the same image acquisition and analysis method, and a total of 2000 cells were counted in hotspot regions for each case. Micro-vessel density (MVD) was calculated by dividing the number of identified micro-vessels by the area under study.

### 2.5. Statistical analysis

Kolmogorov-Smirnov test was employed to determine the normal distribution of all data. Pearson and Spearman correlation tests were employed in data with and without normal distribution, respectively.

The comparisons between T-stage groups, DCE-MRI, and immunohistochemical data were determined with the Mann-Whitney U test.

SPSS 24 software (SPSS Inc, Chicago, IL, USA) was used in the statistical analysis. p < 0.05 was considered statistically significant in all analyzes.

## 3. Results

### 3.1. DCE-MRI data

**Figure 2:**
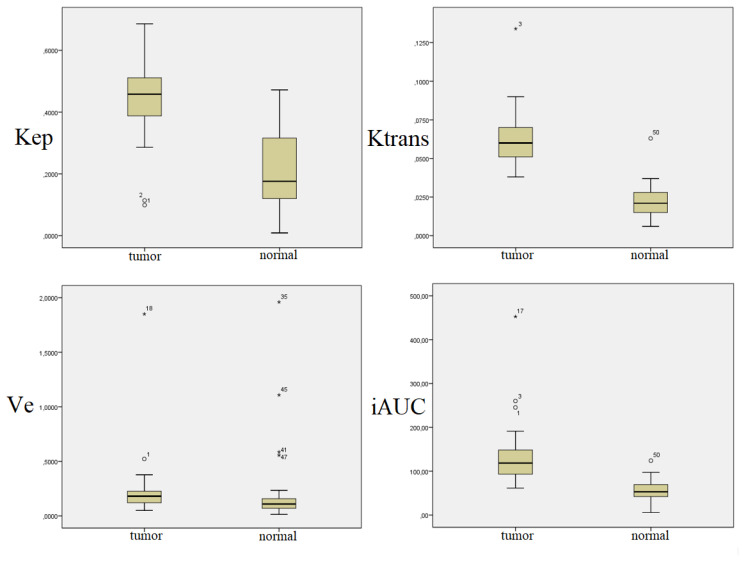
Comparison of DCE-MRI data of tumor and normal tissues.

It was determined that all DCE-MRI parameters measured from tumor tissue were significantly higher when compared to normal tissue (p < 0.01) (Table 1) (Figure 2).

It was determined that there was a positive correlation between Ktrans and iAUC (r = 0.420, p = 0.015), a negative correlation between Kep and Ve (r = −0.843, p = 0.000), a negative correlation between Kep and iAUC (r = −0.488, p = 0.004), and a positive correlation (r = 0.748, p = 0.000) between Ve and iAUC with widest section ROI. There was a positive correlation between Ktrans and Kep (r = 0.399, p = 0.021), a positive correlation between Ktrans and Ve (r = 0.540, p = 0.001), and a positive correlation between Ktrans and iAUC (r = 0.750, p = 0.000) with standard ROI. While there was no correlation between the Kep and other parameters, a positive correlation (r = 0.792, p = 0.000) was determined between Ve and iAUC.

**Figure 3:**
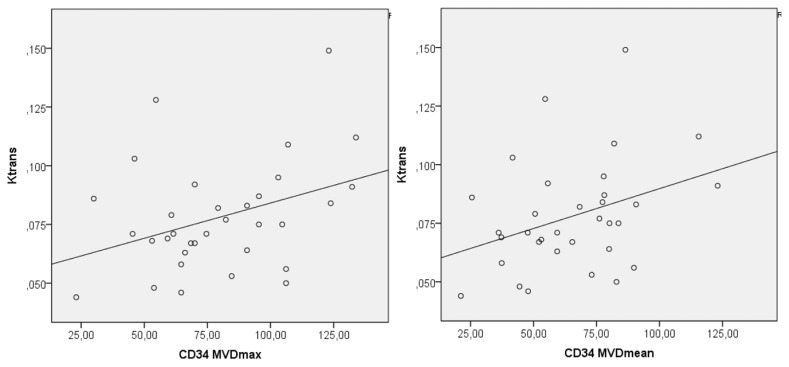
Correlation curve of CD34 MVDmax and CD34 MVDmean parameters with Ktrans (by standard ROI).

No correlation was identified between the DCE-MRI data with the widest section ROI and immunohistochemical analysis. A weak positive correlation was determined between the Ktrans value, which was obtained with standard ROI and CD34 MVDmax and CD34 MVDmean (r = 0.667, p = 0.037 and r = 0.579, p = 0.049, respectively) (Table 2) (Figure 3).

**Figure 4:**
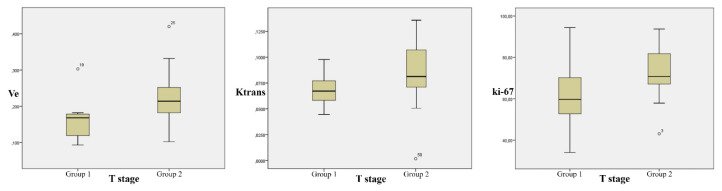
Comparison of Ve, Ktrans, and ki-67 values in Groups 1 and 2.

Based on the T-stage, the mean ki-67 was 61.87 ± 15.92 in Group 1 and it was 72.30 ± 11.49 in Group 2 (p = 0.03). There was no statistically significant difference between the two groups based on MVD parameters (p > 0.05). Ve and Ktrans values (with standard ROI) was statistically significantly higher in Group 2 (p = 0.003 and p = 0.048 respectively) (Figure 4). Furthermore, iAUC value was higher in Group 2; however, the difference was not statistically significant (p = 0.079). No significant difference was determined between the DCE-MRI data with the widest section ROI. Other DCE-MRI parameters did not show statistically significant differences between the groups.

In addition to the above-mentioned findings, the analysis of only the patients who underwent excision surgery demonstrated that the correlation between Ktrans and CD34 MVD was higher (r = 0.667, p = 0.002 for CD34 MVDmax and r = 0.579, p = 0.001 for CD34 MVDmean), and there was a positive correlation between Ktrans and CD105MVD max-mean (r = 0.549, p = 0.018 and r = 0.560, p = 0.016 respectively).

### 3.2. Immunohistochemical data

Immunohistochemical data for tumor tissues are presented in Table 3.

## 4. Discussion

The present study demonstrated that all DCE-MRI parameters were significantly higher in tumor tissue compared to normal tissue. There were also several significant associations between DCE-MRI parameters with immunohistochemical data.

Previously, several studies reported (Chen et al. in 25 patients, Bisdas et al. in 27 patients) that Ktrans, Kep, and Ve parameters were significantly higher in tumor tissue than in normal tissue, similar to our results [13,14]. However, only a few studies investigated the relationships between DCE-MRI and immunohistochemical data in HNSCC. Surov et al. reported a negative correlation between Ve and mean microvascular diameter and between ki-67 and Ktrans in 16 HNSCC patients [11]. The study of Dong et al. evaluating 42 patients with HNSCC concluded that the Ktrans, Ve, and iAUC were significantly higher in poorly differentiated HNSCC patients compared to well-differentiated patients. [1]. In this study, while CD34 MVDmax and CD34 MVDmean values were correlated with Ktrans, no correlation was observed between other DCE-MRI with immunohistochemical parameters. This may be due to the fact that the measurements were made with 2D manual ROI instead of 3D histogram analysis, as in some other studies. Furthermore, another reason for this may be the disruption of blood flow physiology due to vessels of different lengths and diameters in the tumor tissue, which contain tortuous and arteriovenous shunts; and consequently, the presence of high angiogenic activity in areas with low vessel density. It was also reported that MVD measurements depended on the characteristics of the postoperative tissue or biopsy material and different densities could be observed in other areas [10]. As a matter of fact, to eliminate this limitation, only the histopathological data obtained from the excision material were evaluated and it was observed that the correlation between DCE-MRI data and MVD data became stronger. Although the clinical role of MVD in HNSCC is not fully known, the prediction of this immunohistochemical parameter with DCE-MRI data such as Ktrans is still important.

Our study revealed that permeability data of tumor vascularity demonstrated a complex relationship. There are limited studies to draw solid conclusions on the DCE-MRI parameter that can be used to predict immunohistochemical data in HNSCC. In the literature, the most important parameter seems to be Ktrans, which has been associated with poor prognosis and molecular determinants of tumor hypoxia [7,13,15]. Similarly, in our study, ktrans value was found to be significantly higher in tumors with high T stage. However, the correlation between Ktrans and immunohistochemical data could not be clearly defined.

Usually, the vascular and extravascular-extracellular spaces are expected to be proportional. However, this ratio would degrade to provide more oxygen to the tumor tissue; and thus, the Ve parameter would be higher in malignant tumors. We found that Ve values were higher in Group 2, and it was quite promising that the T stage could be predicted with DCE-MRI. In addition, although the iAUC value is not statistically significant, in high-stage tumors found to be higher. In the study of Chen et al. with 42 patients diagnosed with esophageal cancer, according to the histogram analysis of the whole lesion, the value of Ve was found to be significantly higher in T3 tumors compared to T1-T2 tumors [16]. In the study of Huang et al. with 45 patients diagnosed with nasopharyngeal cancer, the relationship between the quantitative and semiquantitative data of DC-MRI obtained from the whole lesion and the tumor stage was examined. In this study, mean Ve and iAUC data were found to be significantly higher in stage T3-T4 tumors compared to T1-T2. There was no significant difference in Ktrans and Kep parameters [17]. In high-stage tumors, neovascularization, perfusion, and permeability between the blood pool and the tumor are higher compared to low-stage tumors. This explains its further development, greater accumulation of contrast agents in the tumor and increased iAUC [10,14]. However, as mentioned above, the semiquantitative methods reflect the signal-intensity curve rather than directly reflecting the contrast agent concentration. These parameters can be easily affected by the MRI protocol and the intrinsic properties of the tissue and are the main restrictive features of the technique. However, the relationship between iAUC and tumor physiology is complicated, and the feature represented by the tumor is not clear since it may be affected by different physiological processes [18].

It has been reported in the literature that ki-67 may be a prognostic factor in various tumors [19]. Although there are conflicting results in HNSCC, there are studies showing the correlation between ki-67 and T stage in laryngeal squamous cell carcinoma [20]. In our research, ki-67 was significantly higher in high-stage tumors compared to low-stage tumors.

### 4.1. Limitations of DCE-MRI technique

DCE-MRI method also has certain limitations. The employment of this technique in HNSCC is in early stages; thus, the technique is not fully standardized yet. Too many factors (such as MRI device, arterial input function measurement method, ROI and parameter selection, pharmacokinetic model variations, etc.) may affect the reliability of the results, which could lead to different results in different institutions [18,21]. Another difficulty in determining the correlations between DCE-MRI parameters and tumor angiogenesis-histology was the tumor heterogeneity at the subvoxel level. In analyzing and interpreting the data, it is necessary to consider the heterogeneity of the tumor. In DCE-MRI studies, user-defined ROIs are generally employed, and this is prone to partial volume average errors. The partial volume average assumes that the kinetic parameters of all pixels are equal, ignoring tumor heterogeneity. Thus, certain authors recommended verifying DCE-MRI imaging parameters with measurements conducted with various 3D ROIs [1,22]. In future studies focusing on optimal DCE-MRI parameters to predict the immunohistochemistry in HNSCC, the ROI method should be used for this parameter and the standard method should be used to verify the data. For this purpose, in 2007, RSNA organized the Quantitative Imaging Biomarkers Alliance® (QIBA) to unite researchers, healthcare professionals, and industry to advance quantitative imaging and the use of imaging biomarkers in clinical trials and clinical practice. QIBA provides guidelines for data collection, analysis, and quality control in DCE-MRI and works on standardization of the technique [23].

### 4.2. Limitations of study

The present study had certain limitations: 1) Thirty-three patients were included. Studies with a higher number of cases are required to improve the results’ accuracy and reveal other possible correlations between DCE-MRI and immunohistochemistry. 2) In certain patients, immunohistochemical analysis was conducted only with biopsy material. Thus, it was likely that DCE-MRI measurements were conducted on sites other than the biopsied area, and as a result, DCE-MRI findings may not fully reflect tumor immunohistochemistry. 3) A 3D histogram analysis covering all tumor sections would provide a more reliable and efficient representation of the whole lesion. However, our workstation did not support the 3D histogram analysis. 4) All HNSCCs were included in our study. However, angiogenesis, tumor behavior, and technical methods may vary in HNSCC; thus, studies focusing on specific subsites may provide more accurate findings. 5) The lack of interobserver variability is another limitation of the study.

DCE-MRI can successfully differentiate the tumor tissue in HNSCC. Furthermore, it was observed that DCE-MRI is a method potentially revealing some immunohistochemical information in vivo. However, its technical development and clinical use are still in the early stages. With standardized image acquisition techniques, data analysis and reporting systems, and multicenter and adequately powered validation studies, the significance of DCE-MRI in HNSCC imaging will become more apparent.

## Figures and Tables

**Table 1:** DCE-MRI data with the widest section ROI.

	Tumor tissue		Normal tissue		P-value
	Mean	Range	Mean	Range	
Ktrans	0.059	0.038–0.135	0.020	0.005–0.063	<0.001
Kep	0.372	0.028–1.431	0.176	0.009–0.442	0.000
Ve	0.181	0.050–1.850	0.107	0.014–1.960	0.006
iAUC	118.54	61.63–452.63	53.18	5.76–124.03	0.000

**Table 2:** Correlation between DCE-MRI and immunohistochemical data.

	Ki-67	CD34 MVDmax	CD34 MVDmean	CD105 MVDmax	CD105 MVDmean
**Ktrans**					
Widest section ROI	r = −0.019* (p = 0.91)	r = 0.152* (p = 0.40)	r = 0.222* (p = 0.21)	r = 0.205* (p = 0.25)	r = 0.242* (p = 0.17)
Standard ROI	r = 0.089** (p = 0.62)	r = 0.364** **(p = 0.037)**	r = 0.346** **(p = 0.049)**	r = 0.236** (p = 0.18)	r = 0.307** (p = 0.08)
**Kep**					
Widest section ROI	r = −160* (p = 0.37)	r = 0.154* (p = 0.19)	r =219* (p=0.22)	r = 0.054* (p = 0.76)	r = 0.109* (p = 0.54)
Standard ROI	r = −0.023* (p = 0.90)	r = 0.235* (p = 0.18)	r = 0.237* (p=0.18)	r = −0.008* (p = 0.96)	r = 0.066* (p = 0.71)
**Ve**					
Widest section ROI	r = 0.124* (p = 0.49)	r = −0.059* (p = 0.74)	r = −0.110* (p = 0.52)	r = 0.019* (p = 0.91)	r = 0.011* (p = 0.95)
Standard ROI	r = 0.060* (p = 0.74)	r = 0.122* (p = 0.50)	r = 0.119* (p = 0.51)	r = 0.245* (p = 0.16)	r = 0.206* (p = 0.25)
**iAUC**					
Widest section ROI	r = 0.191* (p = 0.28)	r = −0.073* (p = 0.68)	r = −0.129* (p = 0.47)	r = 0.047* (p = 0.79)	r = 0.024* (p = 0.89)
Standard ROI	r = 0.092* (p = 0.60)	r = 0.140* (p = 0.43)	r = 0.158* (p = 0.38)	r = 0.278* (p = 0.11)	r = 0.278* (p = 0.11)

* Spearman correlation test;

** Pearson correlation test

**Table 3:** Immunohistochemical data.

Parameter	Mean ± sd	Median	Range
Ki-67 (%)	67.56 ± 14.45	67.70	34.10–94.40
CD34 MVDmean	65.29 ± 23.68	65.38	21.15–123.08
CD34 MVDmax	79.52 ± 28.32	74.61	23.07–133.84
CD105 MVDmean	51.09 ± 24.14	46.46	19.23–115.85
CD105 MVDmax	60.28 ± 27.37	59.23	17.69–128.46
